# Rates and Predictors of Consistent Condom-use by People Living with HIV/AIDS on Antiretroviral Treatment in Uganda

**DOI:** 10.3329/jhpn.v30i3.12290

**Published:** 2012-09

**Authors:** Natal Ayiga

**Affiliations:** Population Research and Training Unit, North West University, Private Bag X2046, Mmabatho 2735, South Africa

**Keywords:** Antiretroviral treatment, Condom-use, PHAs, Uganda

## Abstract

Antiretroviral treatment (ART) has been recognized as one of the methods for reducing the risk of HIV transmission, and access to this is being rapidly expanded. However, in a generalized HIV epidemic, ART could increase unprotected sex by people living with HIV/AIDS (PHAs). This paper assessed the rates and predictors of consistent condom-use by sexually-active PHAs after initiating ART. The study used cross-sectional data on sexual behaviour of 269 sexually-active ART-experienced individuals (95 males and 174 females) aged 18 years and above. The results revealed that 65% (70% of men and 61% of women) used condom consistently after initiating ART. Consistent use of condom was more likely if PHAs had secondary or tertiary-level education and had more than one sex partner in the 12 months preceding the study. However, PHAs were less likely to have used condom consistently if they worked in the informal and formal sectors, belonged to the medium and high-income groups, and were married. PHAs, who were on ART for less than 1 year and 1-2 year(s), had a good self-perception of health, had a sexual partner who was HIV-negative or a partner with unknown HIV status, and desired to bear children, were also less likely to have used condom consistently. The paper concluded that, although the majority of PHAs consistently used condom, there was potential for unprotected sex by PHAs on ART.

## INTRODUCTION

HIV/AIDS continues to be a major global health problem. In 2009, 33.3 million people were living with HIV globally. There were 2.6 million new infections and 1.8 million deaths. Sub-Saharan Africa, with 22.5 million cases, including 1.8 million new infections and 1.3 million deaths, is the most affected region in the world (1). Sub-Saharan Africa also witnessed the highest increase in the number of PHAs on ART from a few thousands in 2003 to more than 3 million by the end of 2008. The increase was substantially higher in countries of Eastern and Southern Africa with high prevalence of HIV/AIDS (2).

The importance of ART in preventing HIV transmission cannot be understated. It has significantly reduced the risk of heterosexual transmissions and transmission from mothers to children (3,4), improved the quality of health of PHAs, reduced their AIDS-related mortality, and improved sexual functions (5). As a result, PHAs live longer and lead healthier, productive and sexually-active lives. These good effects of ART can augment the perception that ART cures AIDS and/or reduces the infectivity of people on ART (6). PHAs may also face difficulty in adhering to a lifetime safe sex because of their desire to bear children (7,8) and consider AIDS as a manageable chronic disease (9). These developments could inadvertently lead to treatment optimism, risk compensation or behavioural disinhibition (10,11), which has created the need for addressing HIV prevention by and among people who are infected.

Studies in developed and developing countries found that the effect of ART on sexual behaviour is mixed. Some studies in developed countries found that ART could trigger unprotected sex in the general population and people of unknown serostatus (12). However, most meta-analytic studies and systematic reviews found that ART does not increase unprotected sex or risky sexual behaviour by PHAs on ART (13-18). Although a few studies in sub-Saharan Africa discovered that some PHAs on ART engaged in unprotected sex due to the perceived non-infectivity or recovery from AIDS by ART (19-21), a number of intervention studies did not find any increase in risk behaviour by PHAs on ART (10,22,23).

Uganda has reduced the HIV/AIDS prevalence to about 6% where it stagnated since 2002 (24) and has made significant progress in expanding access to ART (25). However, more recently, fears that ART could impede consistent use of condom among general population and PHAs have been reported (26). Although some studies in Uganda did not find evidence to suggest that ART could impede condom-use, these studies are few in number and took into account the condom-use at the last sex only (27,28). The objectives of this paper are to assess the rates and predictors of consistent use of condom after initiating ART by sexually-active PHAs in Uganda.

## MATERIALS AND METHODS

### Data and sample

The paper used data from a cross-sectional study conducted in 2005 on PHAs on ART receiving nutrition support provided by World Food Programme (WFP). Although the main objective of collecting the original data was to assess the impact of nutrition support on ART adherence, the survey collected retrospective data on sexual behaviour of PHAs before and after initiating ART. The data used in this paper focused on condom-use behaviour of PHAs after initiating ART.

Data were collected from HIV/AIDS clinics at Nsambya, Hoima, and Soroti hospitals. Nsambya Hospital in Central Uganda is one of the first health facilities to provide ART and social support (including nutrition support) for PHAs in Uganda. Soroti Hospital in Eastern Uganda and Hoima Hospital in Western Uganda collaborate with the Joint Clinical Research Centre, a pioneer AIDS treatment research institution in Uganda to provide routine HIV testing and ART for PHAs. These health facilities were selected because they provided ART and a WFP-supported nutrition programme for PHAs.

The sample for this analysis comprised 45, 96, and 128 individuals from Soroti, Hoima and Nsambya HIV/AIDS clinic respectively. Altogether 269 sexually-active PHAs on ART, including 95 men and 174 women aged 18 years and above, were included in the analysis.

Data were collected through face-to-face exit interviews. The interviews were conducted after healthcare or after the collection of food baskets received on ‘nutrition days’ by PHAs. On these days, PHAs were also provided clinical care and medicines, if required. A pre-tested structured questionnaire translated into local Luganda, Runyankole-Rukiga and Ateso languages, was used for collection of data. All responses were self-reported by the participants.

### Ethical issues

Ethical approval was obtained from the Uganda National Council of Science and Technology (UNCST). Administrative approval was obtained from the District HIV/AIDS Coordinator (DAC) and District Chief Administrative Officer (CAO) who are responsible for HIV/AIDS-related activities in each district where data were collected. Informed consent was obtained from PHAs who voluntarily participated in the study after being explained the objectives and processes of the study. The participants were also assured of their right to refuse or withdraw from the study and that such refusal or withdrawal will not affect their access to ART and nutrition support. Data were also collected from the participants anonymously.

### Measures

The primary outcome measure, the dependent variable of this paper was the consistent use of condom after initiating ART. The condom-use was measured as consistent if sexually-active PHAs reported they always used condom after initiating ART regardless of the duration of ART. Those who never used condom and used condom sometimes were categorized as having used condom inconsistently.

The independent variables comprised sociodemographic characteristics, HIV/AIDS status, and sexual behaviour of PHAs. Sociodemographic data used in this paper include sex, age, place of residence, religion, level of education, occupation, income group, and marital status. The data used for HIV/AIDS include known duration with HIV/AIDS, duration of ART, whether or not ART can reduce risk of HIV infection, current self-perception of health status, and HIV status of usual sex partner categorized as HIV-positive, HIV-negative, and HIV status unknown. Additionally, data on desire to bear children, number of sex partners in the 12 months preceding the study, and frequency of sexual intercourse in the three months preceding the study were used. All the above variables were hypothesized to influence consistent use of condom after initiating ART.

### Data analyses

The statistical analyses were done using the SPSS software. The analyses involved three stages, and the individual was the unit of analysis. The first stage was univariate analysis used for describing the sociodemographic characteristics, HIV/AIDS status, and sexual behaviour of PHAs. At the second stage, the bivariate binary logistic regression was used for investigating the association between consistent use of condom and sociodemographic, HIV/AIDS and sexual behaviour variables. The multivariate analysis was done at the third stage, and it was assumed that the clinic, as a cluster variable, could randomly affect consistent use of condom. This effect of clustering at the clinic level was investigated by using the random intercept logistic regression model (29). Only the variables that were significantly associated with consistent use of condom at the bivariate analysis were included in the final model. A measure of goodness of fit was performed, and the result showed a good fit with p<0.0001 which implies highly significant.

The random intercept logistic regression model was also chosen because the outcome measure was binary where consistent use of condom was coded ‘1’, inconsistent use of condom was coded ‘0’, and the independent variables were all categorical (30). To determine association between consistent use of condom and independent variables, the level of significance (p) was fixed at 0.05.

## RESULTS

### Sociodemographic characteristics

The sociodemographic characteristics of PHAs are presented in Table 1. The table shows that the majority of PHAs (65%) were women. About half of the participants were aged 35 years or above, and the mean age was 36 years for men and 35 years for women. The sample was predominantly urban (87%). Nearly 6 in 10 PHAs were Protestant; 3 in 10 were Catholic; and one in 10 was Musilm. The majority (59%) of participants had no or primary education and, overall, men were better educated than women. Regarding occupation, the majority (61%) worked in the informal sector, 31% worked in the formal sector, and only 8% were students. While men dominated the informal sector, women were the majority in the formal sector. As expected, 59%, mostly women, were in the low-income group, 19% and 22% were in the middle- and high-income group respectively. Overall, 57%, mostly men, were married and 43%, mostly women, were single (never married and formerly married).

### HIV/AIDS and sexual characteristics

Table 2 shows that most PHAs received ART and food support at Nsambya Hospital (48%), followed by Hoima Hospital (36%), and Soroti Hospital (16%). The majority of PHAs knew they were HIV-positive for less than 3 years, and only 14% knew they were HIV-positive for more than 3 years. The perceived mean duration of HIV-positivity was 3.4 years. Men were more likely than women to have known their HIV-positive status for more than 3 years. With regard to the duration of ART, 39% and 37% of PHAs were on ART for less than one year and 1-2 year(s) respectively. Only 24% of PHAs were on ART for more than 2 years, and the mean duration of ART was only 1.6 years for this sample. Men were more likely than women to have been on ART for more than 2 years. Overall, the majority of men (83%) and women (89%) alike believed that ART cannot reduce HIV transmission, and 78% of men and 68% of women perceived their health status as good. With regard to the HIV status of a regular sex partner, the status of 47% of partners was unknown, 32% were HIV-positive, and 21% were HIV-negative. More women (52%) than men (39%) had sex partners of unknown HIV status, and more men than women had an HIV-negative regular sex partner. Table 2 also shows that the majority of men (67%) and women (62%) did not desire to have children.

Data on the number of sex partners in the 12 months preceding the study show that 87% of PHAs had only one sex partner. More women (97%) than men (67%) had only one sex partner in the 12 months preceding the study. Forty-seven percent of PHAs had sex 2-3 times a month,14% had sex only once a month, and 39% had sex only once in the three months preceding the study. Men were more likely than women to have had sex 2-3 times a month. Overall, the majority of PHAs (65%), mostly men, used condom consistently after initiating ART.

### Predictors of consistent use of condoms

Table 3 presents the adjusted and unadjusted odds ratios (ORs) and confidence interval (CI) used for examining the association between consistent condom-use and sociodemographic characteristics, HIV/AIDS status, and sexual behaviour of PHAs. The table shows that PHAs were significantly more likely to have used condom consistently if they had received ART from Hoima (OR 6.36) and Nsambya (OR 4.13) Hospitals, had secondary- or tertiary-level education (OR 2.81), had more than one sex partner in the 12 months preceding the study (OR 4.59), and had sex (regularly) 2-3 times a month (OR 2.39).

**Table. 1.** Percentage distribution of sexually-active ART-experienced people living with HIV/AIDS by selected sociodemographic characteristics disaggregated by sex

Characteristics of respondents	Sex		Total %(n)
	Male % (n)	Female % (n)	
Age (years)			
<35	38 (36)	56 (98)	50 (134)
35+	62 (59)	44 (76)	50 (135)
Mean age	36	35	35.7
Place of residence			
Urban	86 (82)	88 (153)	87 (235)
Rural	14 (13)	12 (21)	13 (34)
Religion			
Protestant	60 (57)	57 (99)	58 (156)
Catholic	27 (26)	34 (60)	32 (86)
Muslim	13 (12)	9 (15)	10 (27)
Level of education			
No/primary	47 (45)	65 (113)	59 (158)
Secondary/Tertiary	53 (50)	35 (61)	41 (111)
Occupation			
Informal sector	64 (61)	60 (104)	61 (165)
Formal sector	26 (25)	33 (58)	31 (83)
Study	10 (09)	7 (12)	8 (21)
Income group			
Low	51 (48)	63 (109)	59 (157)
Medium	23 (22)	17 (30)	19 (52)
High	26 (25)	20 (35)	22 (60)
Marital status			
Currently married	70 (66)	51 (88)	57 (154)
Currently single	30 (29)	49 (86)	43 (115)
Total	35.3 (95)	64.7 (174)	100.0 (269)

However, consistent use of condom by PHAs was significantly less likely if PHAs worked in the informal (OR 0.19) and formal (OR 0.47) sector, belonged to the medium- (OR 0.48) and high-income group (OR 0.42), and were married (OR 0.27). PHAs were also significantly less likely to have used condom consistently if they had been on ART for 1-2 year(s) (OR 0.29), perceived that ART can reduce risk of HIV transmission (OR 0.24), and had a good self-perception of health (OR 0.34). PHAs who had a regular sex partner who was HIV-negative (OR 0.31) or whose HIV status was unknown (OR 0.15), and desired to bear children (OR 0.54), were also significantly less likely to have used condom consistently.

Results of the adjusted random intercept logistic regression model are presented at the bottom of Table 3. The result shows that PHAs who had secondary- or tertiary-level education (OR 3.81) and had more than one sex partner in the 12 months preceding the study (OR 5.07) were significantly more likely to have consistently used condom after initiating ART. However, PHAs who worked in the informal (OR 0.08) and formal (OR 0.21) sectors; belonged to the medium- (OR 0.29) and high-income group (OR 0.31), and were married (OR 0.55), were significantly less likely to have used condom consistently after initiating ART. PHAs who were on ART for less than 1 year (OR 0.33) and 1-2 year(s) (OR 0.11), had a good self-perception of health (OR 0.67), had a partner who was HIV-negative or whose HIV status was unknown (OR 0.16), and desired to bear children (OR 0.43) were also significantly less likely to have used condom consistently after initiating ART.

**Table. 2.** Percentage distribution of sexually-active ART-experienced people living with HIV/AIDS by selected HIV/AIDS characteristics disaggregated by sex

Characteristics of respondents	Sex		Total %(n)
	Male %(n)	Female %(n)	
Health facility			
Soroti Hospital	10 (10)	20 (35)	16 (45)
Hoima Hospital	34 (32)	37 (64)	36 (96)
Nsambya Hospital	36 (53)	43 (75)	48 (128)
Known duration with HIV/AIDS (years)			
At least 2	25 (24)	35 (61)	32 (85)
2-3	44 (42)	59 (103)	54 (145)
>3	31 (29)	6 (10)	14 (39)
Mean duration with HIV (years)	3.5	3.3	3.4
Duration of ART (years)			
<1	28 (60)	44 (77)	39 (104)
1-2	37 (35)	37 (54)	37 (70)
>2	35 (52)	19 (43)	24 (95)
Mean duration of ART (years)	1.7	1.5	1.6
Perception that ART can reduce HIV transmission			
Yes	17 (16)	12 (21)	14 (37)
No	83 (79)	89 (153)	86 (232)
Self-perception of health status			
Good health	78 (74)	68 (118)	71 (192)
Fairly good health	22 (21)	32 (56)	29 (77)
HIV status of regular sex partner			
Positive	31 (29)	33 (58)	32 (87)
Negative	30 (29)	15 (26)	21 (55)
Unknown	39 (37)	52 (90)	47 (127)
Desire to bear children			
Yes	33 (31)	38 (66)	36 (97)
No	67 (64)	62 (108)	64 (172)
Number of sex partners in the past 12 months			
One	67 (64)	97 (169)	87 (233)
Two or more concurrently	33 (31)	3 (5)	13 (36)
Mean number of sex partners	1.3	1.0	1.1
Monthly frequency of sex			
Once a month	14 (13)	14 (25)	14 (38)
2-3 times a month	51 (49)	45 (78)	47 (127)
Very occasional	35 (33)	41 (71)	39 (104)
Mean monthly sexual intercourse	2.2	2.3	2.3
Condom-use			
Consistent	70 (67)	61 (107)	65 (174)
Inconsistent	30 (28)	39 (67)	35 (95)
Total	35.3 (95)	64.7 (174)	100.0 (269)

The result of the test for random effect of clinics as a cluster variable presented at the bottom of Table 3 suggests that clinics did not have any random effect on consistent use of condom by PHAs on ART. This is because the random intercept is −13.8; the residual variance is very low at 0.001; the inter cluster correlation (ICC) is less than 1%; and the likelihood ratio is 0.489 which is not statistically significant.

## DISCUSSION

ART is being increasingly recognized as one of the methods of reducing the risk of HIV transmission. Although increasing access to ART in a generalized HIV epidemic has been greatly supported, this could lead to unprotected sex by PHAs on ART. This view is supported by evidence from both developed and developing countries where unprotected sex by PHAs on ART, with sex partners who are HIV-negative or whose HIV status is not known, has been reported (17,19). This fear has already been raised in Uganda which has a generalized HIV-epidemic and has also greatly expanded access to ART (26). As ART is becoming widely available in high-prevalence societies, more knowledge on the effect of ART on consistent use of condom by PHAs is required to address HIV prevention efforts by and among people who are infected. This study, therefore, investigated rates and predictors of consistent use of condom by sexually-active PHAs after initiation of ART in Uganda.

Overall, this study did not find sufficient evidence to suggest that ART could cause HIV risk compensation through inconsistent use of condom. This is because the study found that the majority of PHAs on ART consistently used condom after initiating ART. This finding agrees with two earlier studies in Uganda (27,28) and a number of meta-analytic studies and systematic reviews in developed and other developing countries (10,14,17,22,27,28). Furthermore, consistent use of condom was not influenced by the clinics the PHAs attended, implying that all three clinics provided AIDS treatment and care, using the same standards prescribed by the HIV/AIDS and the antiretroviral policies in Uganda.

The level of education had the greatest impact on consistent use of condom by PHAs on ART compared to any other indicator in these analyses. Having secondary- or tertiary-level education significantly increased the likelihood of consistent use of condom. This finding is consistent with two earlier studies (31,32). This is likely because of the high self-efficacy for condom-use among people who have secondary- or tertiary-level education (33,34). Educated people are also more likely to be well informed about sexual intercourse as the main route of HIV transmission, making them use condom consistently to prevent transmission of HIV (35).

The study also found that fewer PHAs reported having more than one sex partner in the 12 months preceding the study. Those who reported having more than one sex partner in the reference period were mostly men who were more likely to have used condom consistently. This finding agrees with three previous studies (13,17,18). This result suggests that PHAs wanted to protect their HIV-negative partners and partners with unknown HIV status from infection. It also supports the view that PHAs could have wanted to protect themselves and their HIV-positive partners from re-infection, which agrees with a previous study (36). Consistent use of condom by people with more than one sex partner, whether in the form of serial monogamy or multiple concurrent partnerships (not examined in this analysis), is important in preventing the spread of HIV and re-infection by drug-resistant HIV strains (37). Supporting and reinforcing safe sex in the context of multiple sexual partnerships is particularly important where one of the partners is HIV-positive.

However, this analysis found evidence supporting the view that some PHAs on ART use condom inconsistently. Occupation and level of income are two related factors associated with inconsistent use of condom. PHAs on ART working in the informal sector were less likely to have used condom consistently. This finding is in line with some condom-use studies which found that people working in the informal sector and belonging to the low-income group used condom inconsistently (38). Some studies attributed this to the money or materials used to solicit sex, which may compromise use of condom by people in the low-socioeconomic groups, most of whom are in the informal sector (39). Sex workers and young women have also been found to receive money and other gifts from clients and older sex partners respectively in exchange of unprotected sex (21). Some studies have attributed unprotected sex involving people of the same or different HIV serostatus to the use of alcohol (40).

In Uganda, however, the informal sector includes people in business, who constitute a significant proportion of the middle- and higher-income groups. Before ART was rolled out, most people in the middle- and higher-income brackets, who could afford ART, were engaged in business. It is likely that some of them could have engaged in unprotected sex with their spouses or solicited sex in exchange of money or gifts (39). The concern emanating from this finding is the possibility that some of these PHAs may have had unprotected sex with people who were HIV-negative or whose HIV status was not known, which has serious implications for HIV transmission. This calls for measures that encourage PHAs to use condom consistently.

**Table. 3.** Unadjusted and adjusted odds ratios predicting consistent use of condom by people living with HIV/AIDS on ART in Uganda

Predictor	Unadjusted		Adjusted	
	Odds ratio	95% Cl	Odds ratio	95% Cl
Health facility			
Soroti Hospital	1.00			
Hoima Hospital	6.36****	3.00-13.40		
Nsambya Hospital	4.13***	2.20-7.66		
Sex			
Male	0.77	0.45-1.32		
Female	1.00			
Age (years)			
<35	1.44	0.59-3.52		
35+	1.00			
Place of residence			
Urban	1.03	0.48-2.23		
Rural	1.00			
Religion			
Protestant	0.69	0.26-1.39		
Catholic	0.54	0.22-1.31		
Muslim	1.00			
Educational attainment			
No or primary	1.00	1.49-5.30	1.00	1.63-8.92
Secondary and tertiary	2.81***		3.81***	
Occupation			
Informal sector	0.19***	0.07-0.51	0.08****	0.23-0.33
Formal sector	0.47	0.18-1.26	0.21**	0.06-0.76
Study	1.00		1.00	
Income group			
Low	1.00	0.26-0.90	1.00	0.12-0.66
Medium	0.48*	0.19-0.93	0.29**	0.10-0.92
High	0.42*		0.31*	
Marital Status			
Currently married	0.27****	0.15-0.45	0.55*	0.26-1.11
Currently single	1.00			
Known duration with HIV/AIDS			
At least 2 years	1.94	0.85-4.45		
2-3 years	1.77	0.81-3.91		
More than 3 years	1.00			
Duration on ART (years)			
<1	0.93	0.49-1.74	0.33***	0.14-0.76
1 to 2	0.29***	0.14-0.59	0.11****	0.04-0.29
>2	1.00			
Perception that ART can reduce HIV infection			
Yes	0.24****	0.11-0.49	0.45	0.17-1.18
No	1.00		1.00	
Self-perception of health			
	0.34****	0.19-0.59	0.67*	0.32-1.40
Good health			
	1.00			
Fairly good health			
HIV status of regular partner			
Positive	1.00	0.17-0.57	1.00	0.12-0.61
Negative	0.31****	0.64-0.36	0.27***	0.05-0.53
Unknown	0.15****		0.16***	
Desire to bear children			
Yes	0.54*	0.31-0.64	0.43*	0.19-0.95
No	1.00		1.00	
Number of partners in past 12 months			
One	1.00	1.57-13.43	1.00	1.34-19.22
Two or more	4.59***		5.07**	
Monthly frequency of sex			
Once a month	1.00	1.11-5.10	1.00	0.40-3.35
2-3 times a month	2.39*	0.48-1.49	1.16	0.47-2.00
Once in 3 months	0.85		0.97	
Constant			38.8***	
Cluster/random variable	Effect of clinic as a random variable			
	Random	intercept		-13.8
	Residual	variance		0.001
Clinic	The inter-cluster	correlation	(ICC)	<1%
	Likelihood	ratio (LR) test		p=0.498

Level of significance

*p<0.05;

**p<0.01;

***p=<0.0001;

****p<0.0001;

CI=Confidence interval

Consistent condom-use was also found to be less likely among married PHAs on ART. This finding can be attributed to a number of factors, including partner's objection and inconvenience (41); unwillingness to commit to a lifetime consistent use of condom for reasons, including desire and pressure to bear children (42); and lack of control over sexual decisions and reproductive, economic and social insecurity (43). The perception that condom-use is associated with infidelity could also contribute to the inability to negotiate consistent use of condom in marriage (44). PHAs might have also believed that their usual sex partner was already infected and, therefore, concluded that there was no need to continue using condom. It was also possible that PHAs in marriage had used condom for a long time and might have experienced fatigue in condom-use. Other important barriers to condom-use that have been identified in almost all contexts, including marriage, are stigma and discrimination, and fear of marital and familial instability.

Failure to use condom consistently by married PHAs had implications for transmitting HIV to an HIV-negative partner or a partner of unknown HIV status. A study in Eastern Africa among married people with HIV found that two-thirds had a partner who was uninfected (45). Unprotected sex in marriage could explain the finding by another study that almost half of new HIV infections in Uganda occur within marriage (24). The level of vulnerability to HIV infection in marriage was higher for women because of their subordinate status in relation to men and inability to exert effective control over their sexuality.

The worrying finding in this study is inconsistent condom-use by PHAs on ART, with sex partners who were HIV-negative or whose HIV status was not known. This can occur in several contexts, including marriage, casual sex, and commercial sex. This behaviour has been explained by a feeling of invulnerability, especially if the HIV-negative partner remained uninfected for a long time. This was observed among HIV-negative men who refused to use condom with HIV-positive primary female partners (46). Alcohol and drug-use can also contribute to unprotected sex by PHAs on ART, with HIV-negative partners and partners of unknown HIV status (40). In sub-Saharan Africa, inconsistent condom-use was found to be common in marriage where one of the sex partners was HIV-negative or whose HIV status was unknown (47). This has been attributed to stigma and discrimination associated with disclosure of HIV-positive status (48). Their vulnerability to HIV infection greatly increased where partners with negative and unknown HIV status could not negotiate consistent condom-use because of their young age and being a woman or poor (49). Engaging in unprotected sex with HIV-positive individuals knowingly or unknowingly is a very high-risk sexual behaviour for HIV transmission. This calls for the involvement of PHAs in HIV prevention, which could be effective in changing risk behaviour in generalized HIV epidemics. Involvement of PHAs has the potential for disclosure of fact by HIV-positive individuals, which can encourage their sex partners to test for HIV and ensure that they remain uninfected by consistently using condom with their HIV-positive sex partners.

Inconsistent use of condom by PHAs on ART was also found to be higher among those who had a good self-perception of health. The fact that ART improves the health of PHAs to a level where they become socially productive and sexually active is good but these could be perceived as reduced risk of HIV transmission or even that ART cures AIDS (7). This observation has been reinforced by the finding that PHAs who have been on ART for less than 1 year and 1-2 year(s) were also less likely to have used condom consistently, which agrees with the finding from a previous study (50). These results suggest that ART might have contributed to inconsistent condom-use by PHAs, which could be attributed to the spontaneous processes of sexual activity and the negative effect of condom on sexual satisfaction for PHAs who recently regained their sexual desires. This requires counselling to enable PHAs on ART with necessary skills to use condom consistently as they become healthier and more sexually active.

### Limitations

Although this study has identified some variables that significantly affect consistent use of condom by PHAs on ART, it has some limitations. These include: the small sample-size and purposive selection of study sites; not including PHAs who are not on ART for comparison; self-reporting of condom-use that could have been affected by social desirability bias; and the cross-sectional design which cannot explain changes in condom-use behaviour over time. Nevertheless, analyzing condom-use behaviour of PHAs on ART is very important to understand the main predictors of consistent and inconsistent condom-use. This knowledge is required for developing strategies to prevent HIV transmission by PHAs on ART. It is recommended that studies involving larger samples and PHAs not on ART be conducted in Uganda. The study should use a longitudinal design so that changes in condom-use behaviour in the course of antiretroviral treatment can be examined.

### Conclusions

This study concludes there is no strong reason to suggest that being on ART could lead to HIV risk compensation by adversely affecting consistent use of condom by PHAs in Uganda. This is because the majority of PHAs on ART reported that they consistently used condom after initiating ART. PHAs could have consistently used condom after initiating ART to protect their sex partners from infection. This is likely if PHAs know that their partners are HIV-negative or did not know the HIV status of their sex partners. Conversely, PHAs could have used condom consistently to protect themselves or their HIV-positive partner from re-infection with other strains of HIV. However, the potential for PHAs on ART to engage in unprotected sex has also been observed in one-third of the PHAs analyzed. PHAs who were married, who perceived that ART reduces HIV-infectivity, who had a good self-perception of their own health, and had partners with negative and unknown HIV status used condom inconsistently. The desire to bear children, fatigue in using condom, stigma, and lack of condoms could have contributed the declining trend in inconsistent use of condom in these groups.

It is, therefore, important to acknowledge the aspirations of PHAs and support them in experiencing a satisfying sexual and family life and assist them in adopting and sustaining safe sexual practices. It is recommended that HIV prevention strategies for and by HIV-positive individuals be emphasized in Uganda. These strategies should include counselling on consistent use of condom and provision of free distribution of condoms among PHAs during ART administration.

## ACKNOWLEDGEMENTS

The author most sincerely thanks the WFP Uganda Country Programme for logistical and financial support in collecting the data used in this paper. The author also thanks the PHAs who participated in this study for sharing their experiences. Members of the research staff who participated in the data collection and processing are also greatly appreciated.
